# Supplementary material for: Insights into the hyperglycosylation of human chorionic gonadotropin revealed by glycomics analysis
Source: PLoS One. 2020 Feb 11;15(2):e0228507. doi: 10.1371/journal.pone.0228507 (PMC7012436; doi:10.1371/journal.pone.0228507)
Supplement: S1 Table — Raw data peak picking was performed using Mascot.dll v1.6.0.13 (Applied Biosystems) and Mascot (version 2.6, www.matrixscience.com) was used to search the NCBIProt database for sequences consistent with the MS/MS fragment ions. Peak list generation and database searching were conducted with the default parameters. MS/MS data were used to search the Homo sapiens portion of the NCBIProt protein database with the following parameters: monoisotopic peptide masses, allowing for partial oxidation of methionine residues and carboxymethylation of cysteine residues, mass tolerance of 300 ppm. and fragment ion tolerance of 300 ppm. Tryptic digests of up to 1 missed cleavage were tolerated. Proteins listed contain 3 or more peptides with ion scores higher than 38, which indicates identity or extensive sequence similarity (p<0.05). (PDF) [file pone.0228507.s001.pdf]

S1 Table. NanoLC-MS/MS analysis of hCG-3 GTD11

Raw data peak picking was performed using Mascot.dll v1.6.0.13 (Applied Biosystems) and Mascot (version 2.6, www.matrixscience.com) was used to search the NCBIProt database for sequences consistent with the MS/MS fragment ions. Peak list generation and database searching were conducted with the default parameters. MS/MS data were used to search the *Homo sapiens* portion of the NCBIProt protein database with the following parameters: monoisotopic peptide masses, allowing for partial oxidation of methionine residues and carboxymethylation of cysteine residues, mass tolerance of 300 ppm. and fragment ion tolerance of 300 ppm. Tryptic digests of up to 1 missed cleavage were tolerated. Proteins listed contain 3 or more peptides with ion scores higher than 38, which indicates identity or extensive sequence similarity ($p<0.05$).

Accession (NCBIProt)	Description	Peptides matched	Total Ion Score
1HCN_B	Chain B, HUMAN CHORIONIC GONADOTROPIN	7	503
1HCN_A	Chain A, HUMAN CHORIONIC GONADOTROPIN	4	499
BAF64541.1	immunoglobulin light chain, partial	6	420
NP_001954.2	pro-epidermal growth factor isoform 1 preproprotein	10	345
NP_001638.1	apolipoprotein D precursor	5	298
NP_000884.1	kininogen-1 isoform 2 precursor	8	289
NP_000468.1	serum albumin preproprotein	7	250
NP_004855.2	growth/differentiation factor 15 preproprotein	4	152
NP_008836.2	pregnancy-specific beta-1-glycoprotein 1 isoform 1 precursor	5	150
AAC25485.1	PSGIIA-a	4	136
NP_002265.3	keratin, type I cytoskeletal 13 isoform b	3	147
NP_005214.2	deoxyribonuclease-1 precursor	3	135
NP_000030.1	apolipoprotein A-I isoform 1 preproprotein	3	134
EAX00393.1	glutaminyl-peptide cyclotransferase (glutaminyl cyclase), isoform CRA_b	3	97
