# Supplementary material for: Insights into the hyperglycosylation of human chorionic gonadotropin revealed by glycomics analysis
Source: PLoS One. 2020 Feb 11;15(2):e0228507. doi: 10.1371/journal.pone.0228507 (PMC7012436; doi:10.1371/journal.pone.0228507)
Supplement: S2 Table — Raw data peak picking was performed using Mascot.dll v1.6.0.13 (Applied Biosystems) and Mascot (version 2.6, www.matrixscience.com) was used to search the NCBIProt database for sequences consistent with the MS/MS fragment ions. Peak list generation and database searching were conducted with the default parameters. MS/MS data were used to search the Homo sapiens portion of the NCBIProt protein database with the following parameters: monoisotopic peptide masses, allowing for partial oxidation of methionine residues and carboxymethylation of cysteine residues, mass tolerance of 300 ppm. and fragment ion tolerance of 300 ppm. Tryptic digests of up to 1 missed cleavage were tolerated. Proteins listed contain 3 or more peptides with ion scores higher than 38, which indicates identity or extensive sequence similarity (p<0.05). (PDF) [file pone.0228507.s002.pdf]

S2 Table. NanoLC-MS/MS analysis of hCG-4 GTD10a

Raw data peak picking was performed using Mascot.dll v1.6.0.13 (Applied Biosystems) and Mascot (version 2.6, www.matrixscience.com) was used to search the NCBIProt database for sequences consistent with the MS/MS fragment ions. Peak list generation and database searching were conducted with the default parameters. MS/MS data were used to search the *Homo sapiens* portion of the NCBIProt protein database with the following parameters: monoisotopic peptide masses, allowing for partial oxidation of methionine residues and carboxymethylation of cysteine residues, mass tolerance of 300 ppm. and fragment ion tolerance of 300 ppm. Tryptic digests of up to 1 missed cleavage were tolerated. Proteins listed contain 3 or more peptides with ion scores higher than 38, which indicates identity or extensive sequence similarity ($p<0.05$).

Accession (NCBIProt)	Description	Peptides Matched	Total Ion Score
BAF64541.1	immunoglobulin light chain, partial	10	548
BAC01722.1	immunoglobulin kappa light chain VLJ region, partial	9	533
AMT74553.1	immunoglobulin light chain VRC01c-HuGL, partial	6	360
AMT74548.1	immunoglobulin light chain VRC01c-HuGL, partial	6	354
QEP27269.1	IG c1147_light_IGKV3-20_IGKJ5, partial	4	258
AAW69278.1	anti-tetanus toxoid immunoglobulin light chain variable region, partial	5	240
P00450.1	RecName: Full=Ceruloplasmin; AltName: Full=Ferroxidase; Flags: Precursor	10	377
NP_001638.1	apolipoprotein D precursor	6	330
CAA68763.1	glucan 1, 4-alpha-glucosidase	8	330
NP_001954.2	pro-epidermal growth factor isoform 1 preproprotein	7	307
NP_000884.1	kininogen-1 isoform 2 precursor	6	281
1HCN_B	Chain B, HUMAN CHORIONIC GONADOTROPIN	4	264
1HCN_A	Chain A, HUMAN CHORIONIC GONADOTROPIN	3	254
EAX00393.1	glutaminyl-peptide cyclotransferase (glutaminyl cyclase), isoform CRA_b	4	240
NP_001076.2	alpha-1-antichymotrypsin precursor	4	212
NP_002769.1	prosaposin isoform a preproprotein	5	209
BAB71634.1	unnamed protein product	5	198
ABU90693.2	immunoglobulin lambda 1 light chain, partial	4	164
NP_000053.2	plasma protease C1 inhibitor precursor	5	190
NP_004516.2	low-density lipoprotein receptor-related protein 2 precursor	7	173
NP_001072.2	cubilin precursor	4	171
AAA35692.1	complement cytolysis inhibitor precursor	4	164
NP_000396.2	ganglioside GM2 activator isoform 1 precursor	4	163
NP_005214.2	deoxyribonuclease-1 precursor	3	162
AAA36799.1	uromodulin	4	160
NP_000395.3	beta-galactosidase isoform a preproprotein	4	152
NP_001900.1	cathepsin D preproprotein	3	143
NP_000254.2	alpha-N-acetylglucosaminidase precursor	4	133
EAX00084.1	hCG16977, isoform CRA_c	3	131
NP_000604.1	hemopexin precursor	3	112
