# Supplementary material for: Insights into the hyperglycosylation of human chorionic gonadotropin revealed by glycomics analysis
Source: PLoS One. 2020 Feb 11;15(2):e0228507. doi: 10.1371/journal.pone.0228507 (PMC7012436; doi:10.1371/journal.pone.0228507)
Supplement: S3 Table — Normal distribution test for the difference (Δ) between early (EP-hCG) and late (LP-hCG) pregnancy variables (structural features) using the Shapiro-Wilk statistic. (PDF) [file pone.0228507.s003.pdf]

S3 Table. **Normal distribution for paired samples *t*-test**. Normal distribution test for the difference (Δ) between early (EP-hCG) and late (LP-hCG) pregnancy variables (structural features) using the Shapiro-Wilk statistic.

Tests of Normality

	Shapiro-Wilk		
Variable (str. feature)	Statistic	df	Sig.
ΔLacNAc	.967	3	.650
ΔNeuAc	.999	3	.928
ΔLewisX	.815	3	.152
ΔAgalactosylated	.803	3	.122
Δcore-Fucosylated	.941	3	.532
ΔBisected	.780	3	.068
ΔMono-antennary	.995	3	.868
ΔBi-antennary	.975	3	.697
ΔTri-antennary	.962	3	.624
ΔTetra-antennary	.920	3	.452
ΔHigh Mannose	.790	3	.092
