# Supplementary material for: Insights into the hyperglycosylation of human chorionic gonadotropin revealed by glycomics analysis
Source: PLoS One. 2020 Feb 11;15(2):e0228507. doi: 10.1371/journal.pone.0228507 (PMC7012436; doi:10.1371/journal.pone.0228507)
Supplement: S4 Table — Variables from EP-hCG (E) and LP-hCG samples (L) meeting normal distribution (S1 Table) were subjected to paired samples t-test. Value in bold correspond to variable significant at P<0.05. (PDF) [file pone.0228507.s004.pdf]

S4 Table. **Paired samples *t*-test analysis for EP-hCG and LP-hCG samples**. Variables from EP-hCG (E) and LP-hCG samples (L) meeting normal distribution (**Supplemental Table S1**) were subjected to paired samples *t*-test. Value in bold correspond to variable significant at $P<0.05$.

Paired Samples Test

Pairs of variables (str. features)		Paired Differences Mean	Std. Deviation	Std. Error Mean	95% Confidence Interval of the Difference Lower	Upper
Pair 1	E-LacNAc - L-LacNAc	-5.25333	7.39885	4.27173	-23.63310	13.12644
Pair 2	E-NeuAc - L-NeuAc	9.58667	9.77698	5.64474	-14.70071	33.87404
Pair 3	E-LewisX - L-LewisX	-4.33000	3.02331	1.74551	-11.84032	3.18032
Pair 4	E-Agalac* - L-Agalac	-5.32333	8.71484	5.03152	-26.97220	16.32553
Pair 5	E-Core-F - L-Core-F	-6.28000	12.39481	7.15615	-37.07041	24.51041
Pair 6	E-Bisected - L-Bisected	-21.30667	2.95231	1.70452	-28.64061	-13.97272
Pair 7	E-Mono - L-Mono	8.70000	9.05150	5.22589	-13.78518	31.18518
Pair 8	E-Bi - L-Bi	-5.87667	3.74209	2.16050	-15.17253	3.41920
Pair 9	E-Tri - L-Tri	-3.13000	3.89528	2.24894	-12.80641	6.54641
Pair 10	E-Tetra - L-Tetra	.31000	1.57572	.90974	-3.60431	4.22431
Pair 11	E-HM - L-HM	11.90000	20.29709	11.71853	-38.52076	62.32076

Paired Samples Test

Pairs of variables (str. features)		t	df	Sig. (2-tailed)
Pair 1	E-LacNAc - L-LacNAc	-1.230	2	.344
Pair 2	E-NeuAc - L-NeuAc	1.698	2	.232
Pair 3	E-LewisX - L-LewisX	-2.481	2	.131
Pair 4	E-Agalac - L-Agalac	-1.058	2	.401
Pair 5	E-Core-F - L-Core-F	-.878	2	.473
Pair 6	E-Bisected - L-Bisected	-12.500	2	**.006**
Pair 7	E-Mono- - L-Mono	1.665	2	.238
Pair 8	E-Bi - L-Bi	-2.720	2	.113
Pair 9	E-Tri - L-Tri	-1.392	2	.299
Pair 10	E-Tetra - L-Tetra	.341	2	.766
Pair 11	E-HM - L-HM	1.015	2	.417

* Agalac, Agalactosylated; Core-F, Core-fucosylated; Mono, mono-antennary; Bi, bi-antennary; Tri, tri-antennary; Tetra, tetra-antennary; HM, high mannose.
