# Supplementary material for: Insights into the hyperglycosylation of human chorionic gonadotropin revealed by glycomics analysis
Source: PLoS One. 2020 Feb 11;15(2):e0228507. doi: 10.1371/journal.pone.0228507 (PMC7012436; doi:10.1371/journal.pone.0228507)
Supplement: S5 Table — Variables (structural features) deriving from the EP-hCG and GTD-hCG were subjected to the Shapiro-Wilk statistic. Values in bold correspond to variables significant at P<0.05 (not meeting normal distribution assumption). Note that high mannose (HM) and bi-antennary N-glycans (Bi-) did not meet the normal distribution assumption and therefore they were excluded from further analysis. (PDF) [file pone.0228507.s005.pdf]

**S5 Table**. **Normal distribution test for independent samples *t*-test**. Variables (structural features) deriving from the EP-hCG and GTD-hCG were subjected to the Shapiro-Wilk statistic. Values in bold correspond to variables significant at $P<0.05$ (not meeting normal distribution assumption). Note that high mannose (**HM**) and bi-antennary N-glycans (**Bi-**) did not meet the normal distribution assumption and therefore they were excluded from further analysis.

**Tests of Normality**

| | | Shapiro-Wilk | | |
|---|---|---|---|---|
| Variable (str. feature) | Group | Statistic | df | Sig. |
| **High Mannose** | EP-hCG | .765 | 3 | **.032** |
| | GTD-hCG | .851 | 4 | .230 |
| Mono-antennary | EP-hCG | .995 | 3 | .868 |
| | GTD-hCG | .920 | 4 | .537 |
| **Bi-antennary** | EP-hCG | .755 | 3 | **.011** |
| | GTD-hCG | .869 | 4 | .294 |
| Tri-antennary | EP-hCG | .794 | 3 | .101 |
| | GTD-hCG | .918 | 4 | .528 |
| Tetra-antennary | EP-hCG | .938 | 3 | .521 |
| | GTD-hCG | .830 | 4 | .168 |
| Agalactosylated | EP-hCG | .787 | 3 | .085 |
| | GTD-hCG | .917 | 4 | .519 |
| Core-Fucosylated | EP-hCG | 1.000 | 3 | .985 |
| | GTD-hCG | .809 | 4 | .120 |
| Bisected | EP-hCG | .944 | 3 | .544 |
| | GTD-hCG | .891 | 4 | .388 |
| LacNAc | EP-hCG | .841 | 3 | .217 |
| | GTD-hCG | .943 | 4 | .675 |
| NeuAc | EP-hCG | .988 | 3 | .794 |
| | GTD-hCG | .938 | 4 | .642 |
| LewisX | EP-hCG | .993 | 3 | .845 |
| | GTD-hCG | .953 | 4 | .737 |
