# Supplementary material for: Insights into the hyperglycosylation of human chorionic gonadotropin revealed by glycomics analysis
Source: PLoS One. 2020 Feb 11;15(2):e0228507. doi: 10.1371/journal.pone.0228507 (PMC7012436; doi:10.1371/journal.pone.0228507)
Supplement: S6 Table — Variables from EP-hCG and GTD-hCG samples meeting normal distribution (S3 Table) were subjected to independent samples t-test. Note that mono-antennary (Mono-) and agalactosylated (Agalacto) N-glycans were not found statistically significant (P = 0.402 and P = 0.092 respectively). Values in bold correspond to variables significant at P<0.05 for the equality of variances (var.) and equality of means. (PDF) [file pone.0228507.s006.pdf]

S6 Table. **Independent samples *t*-test analysis for EP-hCG and GTD-hCG samples.** Variables from EP-hCG and GTD-hCG samples meeting normal distribution (**Supplemental Table S3**) were subjected to independent samples *t*-test. Note that mono-antennary (**Mono-**) and agalactosylated (**Agalacto**) N-glycans were not found statistically significant (P=0.402 and P=0.092 respectively). Values in bold correspond to variables significant at P<0.05 for the equality of variances (var.) and equality of means.

Independent Samples Test

		Levene's Test for Equality of Variances		t-test for Equality of Means		
Variable (structural feature)		F	Sig.	t	df	Sig. (2-tailed)
**Mono-antennary**	Equal var. assumed	.710	.438	-.915	5	.402
	Equal var. not assumed			-.835	2.932	.466
Tri-antennary	Equal var. assumed	.353	.578	3.795	5	**.013**
	Equal var. not assumed			3.915	4.872	.012
Tetra-antennary	Equal var. assumed	3.712	.112	2.996	5	**.030**
	Equal var. not assumed			2.631	2.459	.096
**Agalactosylated**	Equal var. assumed	7.057	**.045**	3.125	5	.026
	Equal var. not assumed			2.729	2.398	.092
Core-Fucosylated	Equal var. assumed	2.218	.197	5.235	5	**.003**
	Equal var. not assumed			4.530	2.303	.035
Bisected	Equal var. assumed	.482	.518	6.542	5	**.001**
	Equal var. not assumed			7.064	4.912	.001
LacNAc	Equal var. assumed	.071	.801	6.874	5	**.001**
	Equal var. not assumed			7.207	4.982	.001
NeuAc	Equal var. assumed	.636	.461	-8.421	5	**.001**
	Equal var. not assumed			-9.333	4.545	.000
LewisX	Equal var. assumed	3.775	.110	4.726	5	**.005**
	Equal var. not assumed			4.036	2.177	.049

S6 Table (*continued*). Independent samples *t*-test analysis for EP-hCG and GTD-hCG samples.

Independent Samples Test

Variable (structural feature)		t-test for Equality of Means Mean Difference	Std. Error Difference	95% Confidence Interval of the Difference Lower	Upper
**Mono-antennary**	Equal var. assumed	-4.60333	5.02841	-17.52928	8.32261
	Equal var. not assumed	-4.60333	5.51262	-22.37806	13.17139
Tri-antennary	Equal var. assumed	10.84250	2.85686	3.49870	18.18630
	Equal var. not assumed	10.84250	2.76920	3.66721	18.01779
Tetra-antennary	Equal var. assumed	3.09417	1.03294	.43892	5.74941
	Equal var. not assumed	3.09417	1.17610	-1.16084	7.34918
**Agalactosylated**	Equal var. assumed	18.83583	6.02826	3.33969	34.33198
	Equal var. not assumed	18.83583	6.90195	-6.59952	44.27119
Core-Fucosylated	Equal var. assumed	26.27583	5.01969	13.37232	39.17935
	Equal var. not assumed	26.27583	5.80072	4.21174	48.33992
Bisected	Equal var. assumed	17.19833	2.62872	10.44099	23.95568
	Equal var. not assumed	17.19833	2.43466	10.90616	23.49050
LacNAc	Equal var. assumed	43.60750	6.34409	27.29950	59.91550
	Equal var. not assumed	43.60750	6.05074	28.03655	59.17845
NeuAc	Equal var. assumed	-52.16833	6.19531	-68.09387	-36.24279
	Equal var. not assumed	-52.16833	5.58966	-66.98012	-37.35654
LewisX	Equal var. assumed	8.56417	1.81226	3.90561	13.22272
	Equal var. not assumed	8.56417	2.12182	.11048	17.01785
