# Supplementary material for: Insights into the hyperglycosylation of human chorionic gonadotropin revealed by glycomics analysis
Source: PLoS One. 2020 Feb 11;15(2):e0228507. doi: 10.1371/journal.pone.0228507 (PMC7012436; doi:10.1371/journal.pone.0228507)
Supplement: S1 Fig — MALDI-TOF MS spectra of permethylated N-glycans derived from (A) EP-hCG2, (B) LP-hCG2, (C) EP-hCG3 and (D) LP-hCG3 samples. Structures above a bracket were not unequivocally defined. Red peaks correspond to bisected N-glycans with various antenna configurations. (C, D) Horizontal line above the spectra indicates the area and the level of zoom. Putative structures are based on composition, tandem MS, β-galactosyltransferase experiment and knowledge of biosynthetic pathways. All molecular ions are [M+Na]+. (PDF) [file pone.0228507.s007.pdf]

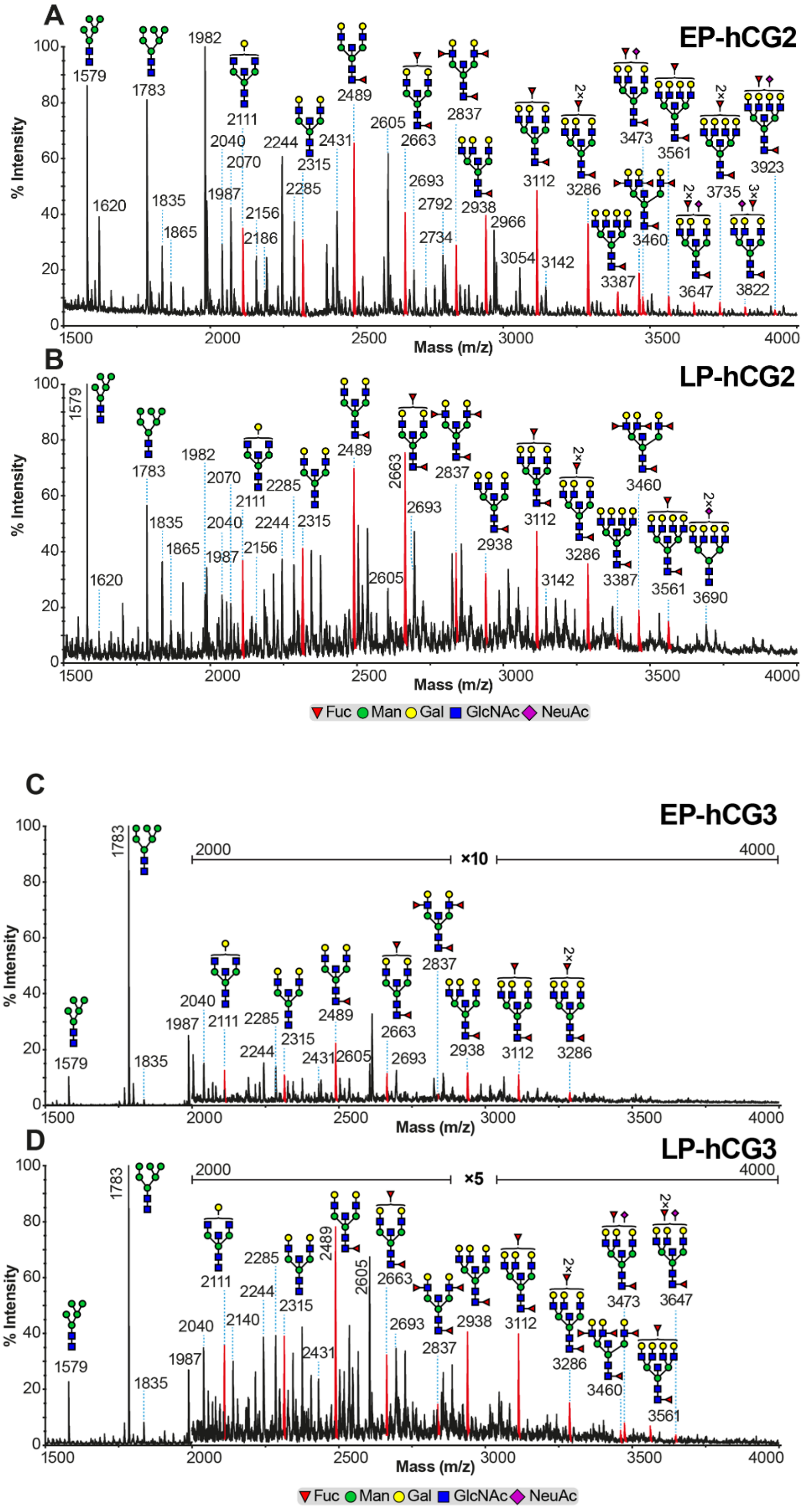

S1 Fig. **N-glycomic profiles of hCG samples**. MALDI-TOF MS spectra of permethylated N-glycans derived from (**A**) EP-hCG2, (**B**) LP-hCG2, (**C**) EP-hCG3 and (**D**) LP-hCG3 samples. Structures above a bracket were not unequivocally defined. Red peaks correspond to bisected N-glycans with various antenna configurations. (**C**, **D**) Horizontal line above the

spectra indicates the area and the level of zoom. Putative structures are based on composition, tandem MS, β-galactosyltransferase experiment and knowledge of biosynthetic pathways. All molecular ions are $[M+Na]^+$.
