# Supplementary material for: Insights into the hyperglycosylation of human chorionic gonadotropin revealed by glycomics analysis
Source: PLoS One. 2020 Feb 11;15(2):e0228507. doi: 10.1371/journal.pone.0228507 (PMC7012436; doi:10.1371/journal.pone.0228507)
Supplement: S2 Fig — MALDI-TOF MS spectra of permethylated N-glycans derived from (A) GTD-hCG2, (B) GTD-hCG3 and (C) GTD-hCG4 samples. Structures above a bracket were not unequivocally defined. Putative structures are based on composition, tandem MS. All molecular ions are [M+Na]+. (PDF) [file pone.0228507.s008.pdf]

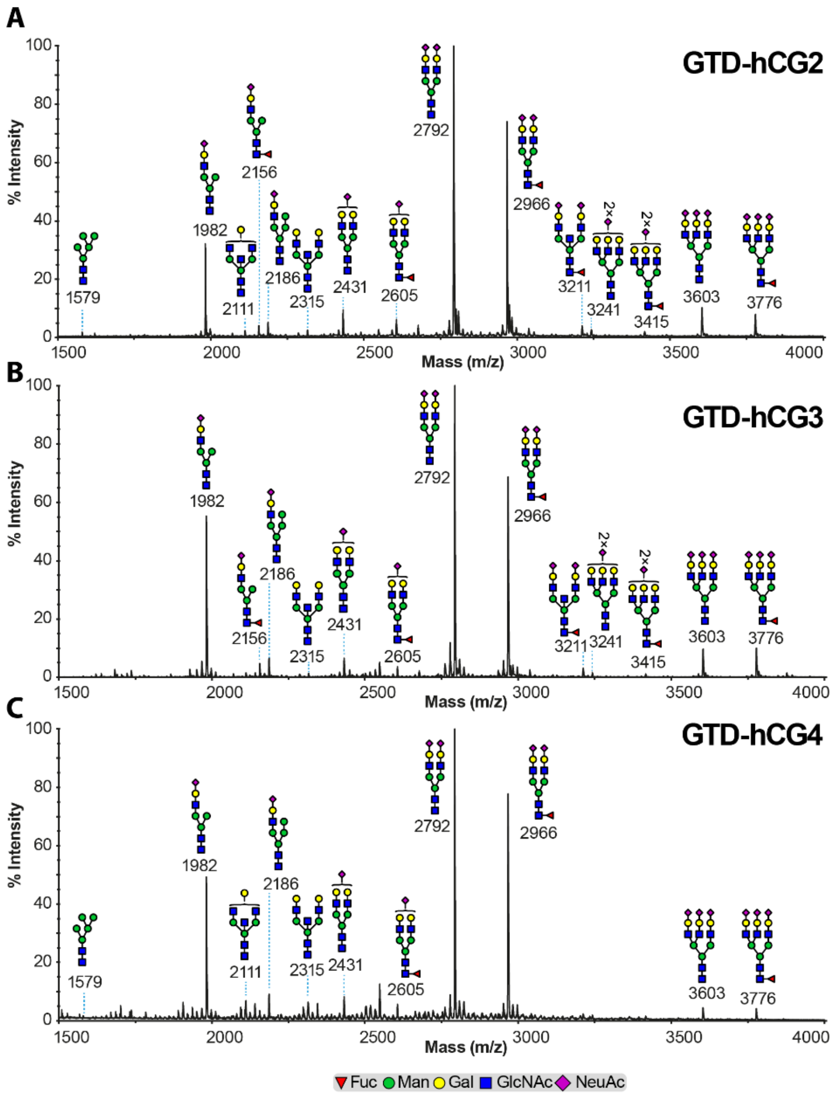

S2 Fig. N-glycomic profiles of GTD-hCG samples. MALDI-TOF MS spectra of permethylated N-glycans derived from (**A**) GTD-hCG2, (**B**) GTD-hCG3 and (**C**) GTD-hCG4 samples. Structures above a bracket were not unequivocally defined. Putative structures are based on composition, tandem MS. All molecular ions are $[M+Na]^+$.
