# Supplementary material for: Insights into the hyperglycosylation of human chorionic gonadotropin revealed by glycomics analysis
Source: PLoS One. 2020 Feb 11;15(2):e0228507. doi: 10.1371/journal.pone.0228507 (PMC7012436; doi:10.1371/journal.pone.0228507)
Supplement: S3 Fig — Representative MALDI-TOF/TOF MS/MS spectra of the molecular ions found at (A, B) m/z 3286 and (C, D) 3460 for the EP and LP-hCG1 samples respectively. Horizontal dashed lines correspond to indicated losses from the molecular ion [M+Na]+. For (A and B), the fragment ion at m/z 3080 corresponds to the elimination of a fucose residue from the molecular ion at m/z 3286, indicative of a fucose residue being on the C3 of GlcNAc residue (Lewis-X). Similarly, for (C and D), the fragment ion at m/z 3254, corresponds to the elimination of a fucose residue from the molecular ion at m/z 3460. (PDF) [file pone.0228507.s009.pdf]

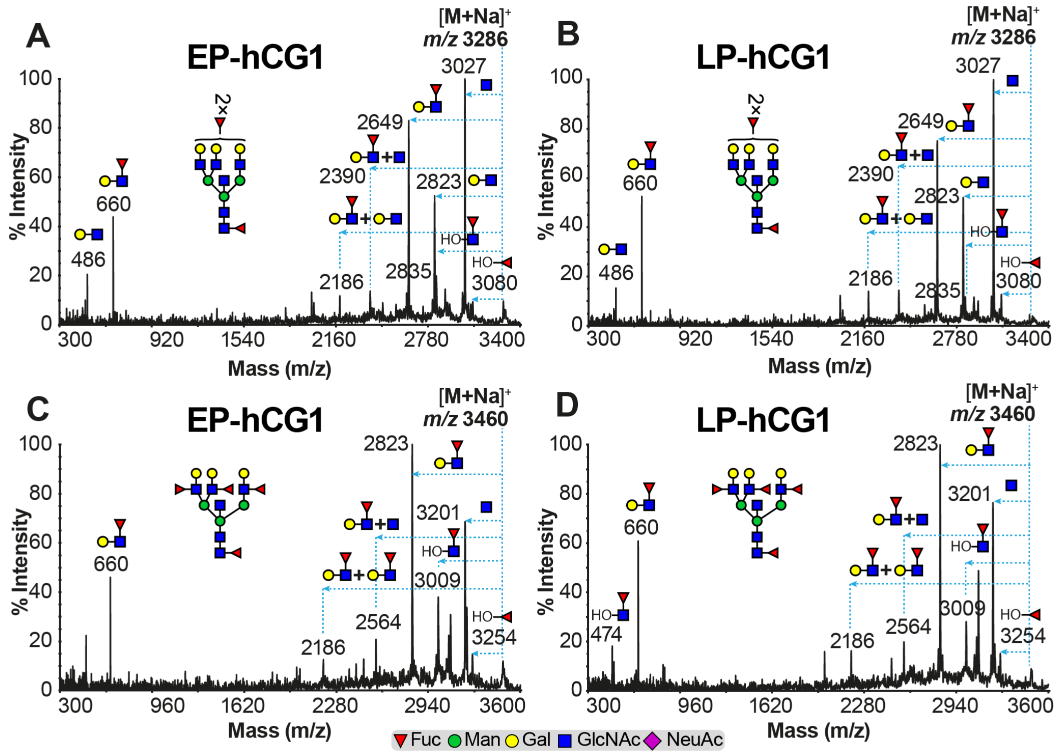

S3 Fig. EP and LP hCG samples contain Lewis^{X} antigens. Representative MALDI-TOF/TOF MS/MS spectra of the molecular ions found at (**A**, **B**) *m/z* 3286 and (**C**, **D**) 3460 for the EP and LP-hCG1 samples respectively. Horizontal dashed lines correspond to indicated losses from the molecular ion $[M+Na]^{+}$. For (A and B), the fragment ion at *m/z* 3080 corresponds to the elimination of a fucose residue from the molecular ion at *m/z* 3286, indicative of a fucose residue being on the C3 of GlcNAc residue (Lewis-X). Similarly, for (C and D), the fragment ion at *m/z* 3254, corresponds to the elimination of a fucose residue from the molecular ion at *m/z* 3460.
