# Supplementary material for: Insights into the hyperglycosylation of human chorionic gonadotropin revealed by glycomics analysis
Source: PLoS One. 2020 Feb 11;15(2):e0228507. doi: 10.1371/journal.pone.0228507 (PMC7012436; doi:10.1371/journal.pone.0228507)
Supplement: S4 Fig — MALDI-TOF MS spectra of permethylated N-glycans derived from (A) EP-hCG1 sample (top panel, control, same as Fig 1A; lower panel, after GalT) and (B) LP-hCG1 sample (top panel, control, same as Fig 1B; lower panel, after GalT). Note the molecular ions at m/z 2315, 2489, 2663, 2837, 2938, 3112, 3286 3460, 3473 and 3647 on EP-hCG1 and LP-hCG1 spectra before the GalT experiment (A and B, top panels), which correspond to complex mature N-glycans with a single terminal GlcNAc residue. The same ions were also detected on the EP-hCG1 and LP-hCG1 spectra after the GalT experiment (A and B, lower panels), indicating that those terminal GlcNAc residues are not subject to a GalT experiment. Therefore, the above molecular ions correspond to bisected N-glycan structures. On the contrary, on EP-hCG1 and LP-hCG1 spectra before the GalT experiment (A and B, top panels), the molecular ions at m/z 1835 and 1865 correspond to agalactosylated N-glycans (GlcNAc terminated residues on mannose arms). These ions are not present in GalT samples (A and B lower panels) indicating that these ions are subject to the β-galactosyltransferase experiment further indicating that they do not correspond to bisected N-glycans. Putative structures are based on composition, tandem MS and knowledge of biosynthetic pathways. All molecular ions are [M+Na]+. (PDF) [file pone.0228507.s010.pdf]

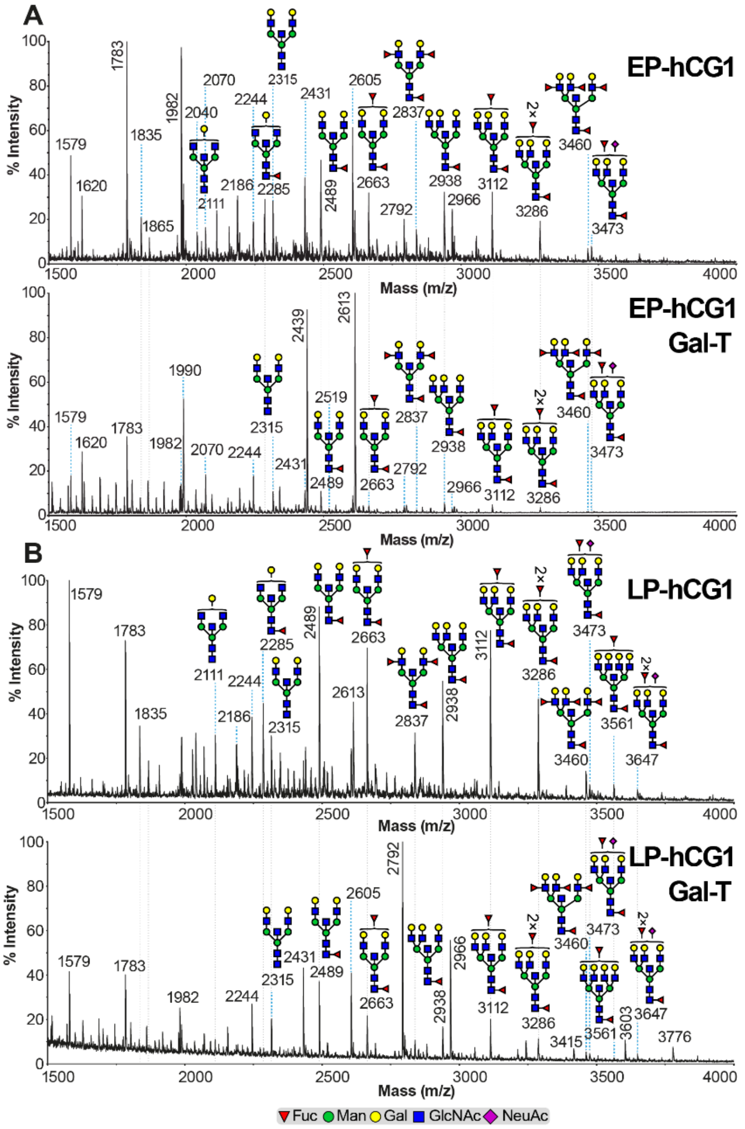


**S4 Fig. β-Galactosyltransferase (GalT) experiment on hCG1 samples**. MALDI-TOF MS spectra of permethylated N-glycans derived from (**A**) EP-hCG1 sample (top panel, control, same as **Fig. 1A**; lower panel, after GalT) and (**B**) LP-hCG1 sample (top panel, control, same as **Fig. 1B**; lower panel, after GalT). Note that on EP-hCG1 and LP-hCG1 spectra before the GalT experiment (**A** and **B**, top panels), the molecular ions at *m/z* 2315, 2489, 2663, 2837, 2938, 3112, 3286 3460, 3473 and 3647 which correspond to complex mature N-glycans with a single terminal GlcNAc residue. The same ions were also detected on the EP-hCG1 and LP-hCG1 spectra after the GalT experiment (**A** and **B**, lower panels), indicating that those terminal GlcNAc residues are not subject to a GalT experiment. Therefore, the above molecular ions correspond to bisected N-glycan structures. On the contrary, on

EP-hCG1 and LP-hCG1 spectra before the GalT experiment (**A** and **B**, top panels), the molecular ions at *m/z* 1835 and 1865 correspond to agalactosylated N-glycans (GlcNAc terminated residues on mannose arms). These ions are not present in GalT samples (**A** and **B** lower panels) indicating that these ions are subject to the β-galactosyltransferase experiment further indicating that they do not correspond to bisected N-glycans. Putative structures are based on composition, tandem MS and knowledge of biosynthetic pathways. All molecular ions are $[M+Na]^+$.
