# Supplementary material for: Insights into the hyperglycosylation of human chorionic gonadotropin revealed by glycomics analysis
Source: PLoS One. 2020 Feb 11;15(2):e0228507. doi: 10.1371/journal.pone.0228507 (PMC7012436; doi:10.1371/journal.pone.0228507)
Supplement: S5 Fig — Partial MALDI-TOF MS spectra of permethylated N-glycans derived from EP-hCG, LP-hCG and GTD-hCG samples as indicated on the corresponding spectra. Red peaks correspond to paucimannose type N-glycans. For comparison of the relative intensity of the paucimannose type N-glycans compared to the relative intensity of molecular ions as shown in Fig 1 and S1 Fig, compare the ratio of paucimannose type N-glycans to the high mannose N-glycans found at m/z 1579 and 1783. All molecular ions are [M+Na]+. (PDF) [file pone.0228507.s011.pdf]

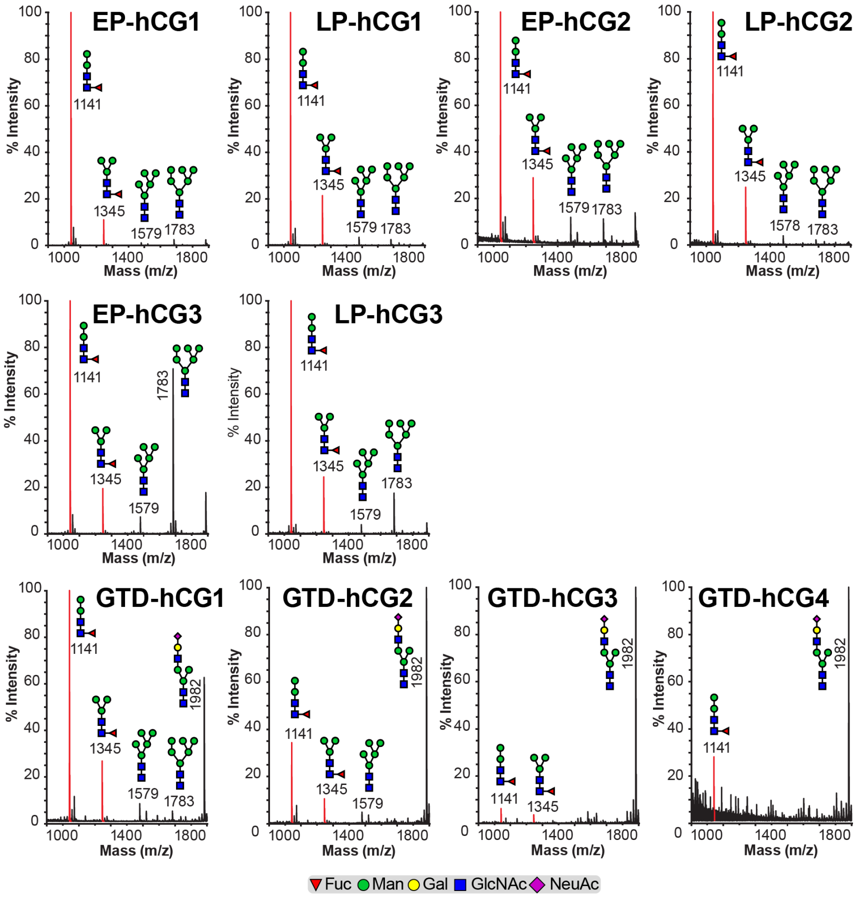

S5 Fig. Paucimannose type N-glycans on hCG samples. Partial MALDI-TOF MS spectra of permethylated N-glycans derived from EP-hCG, LP-hCG and GTD-hCG samples as indicated on the corresponding spectra. Red peaks correspond to paucimannose type N-glycans. For comparison of the relative intensity of the paucimannose type N-glycans compared to the relative intensity of molecular ions as shown in **Fig. 1** and **Supplemental Fig. 1**, compare the ratio of paucimannose type N-glycans to the high mannose N-glycans found at *m/z* 1579 and 1783. All molecular ions are $[M+Na]^+$.
