# Supplementary material for: Insights into the hyperglycosylation of human chorionic gonadotropin revealed by glycomics analysis
Source: PLoS One. 2020 Feb 11;15(2):e0228507. doi: 10.1371/journal.pone.0228507 (PMC7012436; doi:10.1371/journal.pone.0228507)
Supplement: S6 Fig — MALDI-TOF MS spectra of permethylated N-glycans derived from (A) EP-hCG4 and (B) LP-hCG4 samples. Putative structures are based on composition, tandem MS. All molecular ions are [M+Na]+. (PDF) [file pone.0228507.s012.pdf]

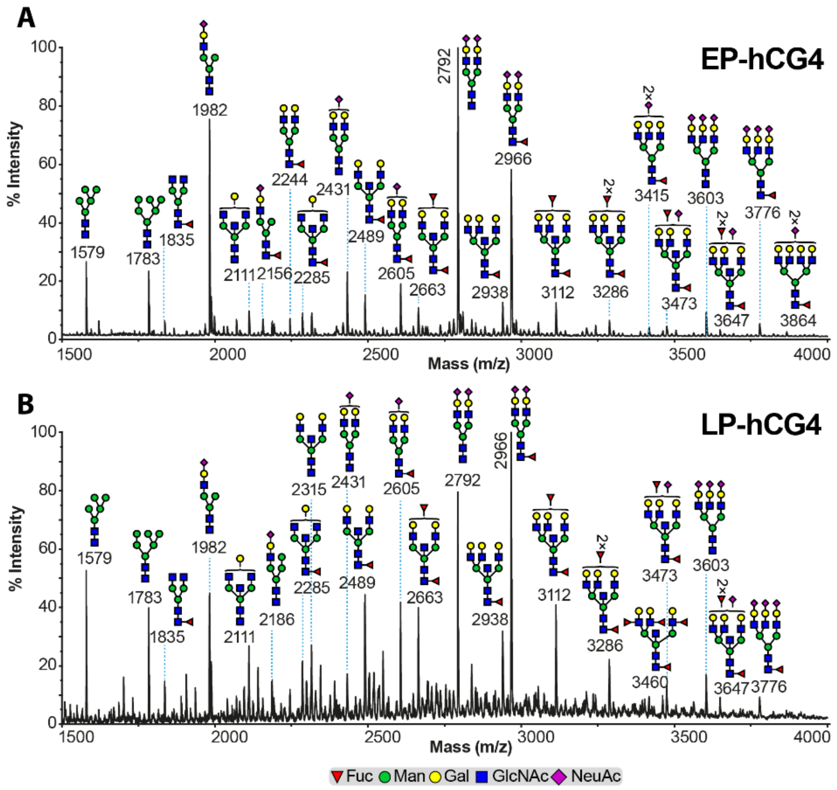

S6 Fig. N-glycomic profiles of pre-eclampsia samples. MALDI-TOF MS spectra of permethylated N-glycans derived from (**A**) EP-hCG4 and (**B**) LP-hCG4 samples. Putative structures are based on composition, tandem MS. All molecular ions are $[M+Na]^+$.
