# Supplementary material for: Insights into the hyperglycosylation of human chorionic gonadotropin revealed by glycomics analysis
Source: PLoS One. 2020 Feb 11;15(2):e0228507. doi: 10.1371/journal.pone.0228507 (PMC7012436; doi:10.1371/journal.pone.0228507)
Supplement: Attachment — Submitted filename: Response to Reviewers..docx [file pone.0228507.s013.docx]

**Insights into the hyperglycosylation of human chorionic gonadotropin revealed by glycomics analysis (PONE-D-19-25594)-Response to reviewers**

Reviewer 1 : Specific points have been addressed as follows:

1) *It would be very useful to show the measured proteomes of representative hCG samples. This would demonstrate the extent to which observed released glycans can be attributed to hCG, rather than co-isolated glycoproteins.*

Representative proteomic data has been added to the supporting information section and cited in the manuscript.

2) *Please discuss the rationale for using MALDI TOF MS profiling of glycans. LC-MS has better dynamic range and would be the logical choice for quantitative studies.*

The use of MALDI TOF MS for profiling of glycans is a highly effective methodology. Whilst we agree with the reviewer that in fields such as proteomics LC-MS has indeed been almost universely adopted as the MS method of choice in the glycan analytic field there is a greater diversity of MS methodology utilized. For example, a simple pubmed search with the terms MALDI-MS glycan 2019 gives 109 publications whilst LC-MS glycan 2019 gives 104 publications. We have also added the following text into the paper to allow a greater explanation of our MALDI-MS based methodology.

“Τhe N-glycan profiles of all hCG samples derived from the above pregnancy conditions were determined using a glycomics strategy previously optimised for the characterisation of N-glycomes from a wide variety of samples including pregnancy associated glycoproteins, such as human glycodelin (41). This glycomic strategy is based on MALDI-MS and MS/MS analyses of the total population of N-glycans after their release from the polypeptide backbone of hCG via digestion with peptide N-glycosidase F. The glycans were permethylated prior to MALDI analysis in order to enhance sensitivity and to allow unambiguous MS/MS fragmentation. The MALDI-MS experiments defined glycan compositions whilst MS/MS experiments determined antennae sequences, as well as the location of fucose residues in Lewis structures”

3) *How is “ultrasensitive” MALDI distinguished from traditional MALDI?*

We have removed the term “ultrasensitive” throughout the manuscript.

4) *It does not appear that the authors released O-glycans. Doing so would have allowed them to shed light on the extent to which hyperglycosylation of hCG is due to changes in O-glycosylation as mentioned on p. 3, line 74.*

We agree with the reviewer that it would indeed have been advantageous to characterise both the N- and O-glycosylation of the hCG samples. However, sample quantities were very limited reflective of the challenges associated with obtaining the original starting biological material from pregnant women. This does not detract from our original objective which was to characterise the poorly understood N-glycosylation of pregnancy hCG. In so doing we have made important new discoveries.

5) *The quantitative interpretation of the MS data depends on the reproducibility of the method employed. On line 152 the text states that permethylated glycan samples were dissolved in 10 microliter of solvent. What measures were taken to keep the glycan concentration constant among the samples? How many shots were acquired per summed MALDI MS spectrum? How many technical replicates were acquired? What is the reproducibility of the HCG purification, digestion, glycan release, workup method? What sample was used as a performance standard to verify consistent glycan quantification?*

Many of these questions are not as relevant in glycomic analysis compared to proteomic analysis. All relevant experimental details are available in the materials and methods section of the paper and the reference publications. Our glycan quantification relies on comparing relative abundance of ions within a spectrum. In glycomics experiments it has been demonstrated that quantitations based on signal intensities of permethylated glycans with in the MALDI-TOF MS spectrum is a reliable method (see Wada, Y., Azadi, P., Costello, C. E., Dell, A., Dwek, R. A., Geyer, H., Geyer, R., Kakehi, K., Karlsson, N. G., Kato, K., Kawasaki, N., Khoo, K. H., Kim, S., Kondo, A., Lattova, E., Mechref, Y., Miyoshi, E., Nakamura, K., Narimatsu, H., Novotny, M. V., Packer, N. H., Perreault, H., Peter-Katalinic, J., Pohlentz, G., Reinhold, V. N., Rudd, P. M., Suzuki, A., and Taniguchi, N. (2007) Glycobiology 17, 411-422).

6) *As stated in the abstract and elsewhere, the authors seem to use a p value threshold to identify glycan abundance differences that are significant. Either the p values or the significance threshold should be corrected for multiple comparisons. Please confirm the method used for correction.*

We have added the following text into the paper to allow a greater explanation.

“All statistical analyses were performed using SPSS version 25. A paired *t*-test was applied to analyse differences for paired hCG samples (EP-hCG versus LP-hCG), while an independent sample *t*-test was used for differences between EP-hCG and GTD-hCG. Significance was taken as *P* < 0.05. The significance threshold was corrected for multiple comparisons using Bonferroni method. For the paired *t*-test epitopes variables (LacNAc, NeuAc and Lewis^X^), the corrected *P* was set at <0.0166, while for the N-glycans variable (high mannose, mono-, bi-, tri- and tetra-antennary N-glycans, agalactosylated, bisected and core-fucosylated N-glycans) it was set at <0.0063. For the independent sample *t*-test epitopes variables (LacNAc, NeuAc and Lewis^X^), the corrected *P* was set at <0.0166, while for the N-glycans variable (mono-, tri- and tetra-antennary N-glycans, agalactosylated, bisected and core-fucosylated N-glycans) it was set at <0.0083. The Bejamini and Hochberg method for false discovery rate was also applied. The method was available in the SPSS software package through the syntax command following instructions published on the IBM support web page (Bejamini and Hochberg, document number 418001). The strictest result of the above methods was taken into account in terms of corrected significance. For the paired *t*-test, the test of normality with Shapiro-Wilk’s statistic and outliers was taken into consideration for the values resulting from the differences of the above variables (EP-hCG versus LP-hCG). For the independent sample *t*-test, the test of normality with Shapiro-Wilk’s statistic, outliers, and variance according to Levene’s test for equality of variances were taken into consideration. Variables not meeting the above assumptions were excluded from the analysis.

Hierarchical cluster analysis was applied using between-groups linkage as a clustering method and Euclidean distance as a measure interval (similar results were obtained with other clustering methods and measure intervals). On EP-hCG versus GTD-hCG samples cluster analysis was performed for the variables found to be significant after correction for multiple comparisons using the criteria as stated above (core fucosylation, bisected, LacNAc, Lewis^X^, NeuAc). For the complete set of samples (EP-hCG, LP-hCG and GTD-hCG) hierarchical cluster analysis was performed using as variables the NeuAc and bisected N-glycans.”

Reviewer 2 : Specific points have been addressed as follows:

1) *MALDI is not generally sensitive at least compared to nanoESI so calling it “ultrasensitive MALDI” seems a little deceiciving. It doesn’t seem like they modified the instrument, so it’s a standard MALDI.*

We have removed the term “ultrasensitive” throughout the manuscript.

2) *The sample size is really small. There were four subjects and one was dropped. The statistical analysis seems like an overkill, given the sample size. Some comment here regarding the sample size would be welcome.*

Please see responses to points 4 and 6 of reviewer 1.

3) *What is the general CV of the MALDI method? There were no reproducibility studies performed or cited.*

Please see response to points 5 reviewer 1.

4) *The structures are all mainly putative, and there is too much emphasis on the structures given that they are guesses. For example, can they confirm that these are really Lewis x structures? The structural guesses should be toned down a bit.*

We apologies for not making it clearer how are glycan structures are defined. The structural assignments that we have presented are rigorous. To allow a greater understanding to the general reader we have added the following text into the paper to allow a greater explanation.

“Τhe N-glycan profiles of all hCG samples derived from the above pregnancy conditions were determined using a glycomics strategy previously optimised for the characterisation of N-glycomes from a wide variety of samples including pregnancy associated glycoproteins, such as human glycodelin (41). This glycomic strategy is based on MALDI-MS and MS/MS analyses of the total population of N-glycans after their release from the polypeptide backbone of hCG via digestion with peptide N-glycosidase F. The glycans were permethylated prior to MALDI analysis in order to enhance sensitivity and to allow unambiguous MS/MS fragmentation. The MALDI-MS experiments defined glycan compositions whilst MS/MS experiments determined antennae sequences, as well as the location of fucose residues in Lewis structures.”

5) *Similarly, the structure of the bisecting GlcNAc. There are some comments regarding transferases, but it’s not clear nor is it shown that these can determine the structure.*

We have added the following text into the paper to allow a greater explanation.

“Evidence for bisected glycans was obtained from observing whether glycans which had been shown by MS/MS to contain terminal GlcNAc residues were capped with a galactose residue after treatment with β-galactosyltransferase and UDP-Gal. Bisected GlcNAc residues are not accessible to β-galactosyltransferase and therefore are not capped with a galactose residue during this procedure. In contrast, terminal GlcNAc residues on truncated N-glycan antennae are fully accessible to the β-galactosyltransferase. Therefore, their capping with galactose serves as a positive control for β-galactosyltransferase activity. This methodology has been previously shown to be a reliable way of confirming bisected GlcNAc (38).”
